# Identification of secretory protein related biomarkers for primary biliary cholangitis based on machine learning and experimental validation

**DOI:** 10.1515/biol-2025-1192

**Published:** 2025-12-30

**Authors:** Zihao Xu, Yue Cai, Yifan Liu, Jun Xu, Sheng Guo, Lihan Zhou, Yang Ji, Lei Zhan, Liangbin Cheng

**Affiliations:** School of Chinese Medicine, Hubei University of Chinese Medicine, Wuhan, Hubei, 430065, China; Hubei Provincial Hospital of Traditional Chinese Medicine, Wuhan, Hubei, 430061, China; Hubei University of Traditional Chinese Medicine, Wuhan, Hubei, 430065, China

**Keywords:** biomarkers, bioinformatics, machine learning, secretory proteins, primary biliary cholangitis

## Abstract

Primary biliary cholangitis (PBC) is a chronic autoimmune liver disease that causes bile duct damage, liver fibrosis, and cirrhosis, significantly affecting patients’ lives and healthcare costs. Early diagnosis is critical but is hindered by the limited sensitivity of existing biomarkers, particularly in patients who are negative for anti-mitochondrial antibodies. This limitation underscores the need for more reliable biomarkers. Our study focuses on secretory proteins as potential diagnostic biomarkers and aims to elucidate gene expression profiles associated with PBC using bioinformatics methods and machine learning. We identified 827 downregulated and 639 upregulated genes related to mitochondrial function and immune pathways. Additionally, Weighted Gene Co-expression Network Analysis revealed a blue module comprising 1,949 genes linked to PBC. Machine learning identified between 14 and 18 key diagnostic genes. Using a Gaussian Mixture Model, we achieved an area under the curve of 0.96, indicating excellent diagnostic performance. Notable genes included the upregulated CSF1R, PLCH2, and SLC38A1, as well as the downregulated CST7. Animal experiments further supported these bioinformatics findings. Our research highlights secretory proteins as promising biomarkers for the early diagnosis of PBC, with potential applications in developing precise diagnostic tools and personalized therapies. This work paves the way for future studies involving larger cohorts and multi-omics data.

## 1 Introduction

Primary biliary cholangitis (PBC) is a chronic autoimmune liver disease characterized by the progressive destruction of small bile ducts, leading to cholestasis and potential liver cirrhosis [1]. Epidemiological data indicate an incidence of approximately 2–5 cases per 100,000 individuals per year, with a female-to-male ratio of about 9:1, predominantly affecting women over 40 years of age. This high prevalence among middle-aged women underscores an urgent need for improved diagnostic and therapeutic strategies. The disease is often associated with the presence of anti-mitochondrial antibodies (AMAs) [2]; however, a significant proportion of patients exhibit AMA-negative PBC, which complicates diagnosis and treatment due to the absence of a key diagnostic marker. Current diagnostic strategies primarily rely on biochemical markers, liver function tests, and liver biopsies; yet these methods fall short in early detection and risk stratification [3].

Despite the identification of potential biomarkers such as ficolin-1, CD40, and hexokinase-1, their clinical utility remains limited due to limitations such as insufficient sensitivity and specificity, as well as lack of validation across diverse patient populations. In particular, these biomarkers have not fully addressed the diagnostic challenges posed by AMA-negative PBC. Therefore, this study focuses on secreted proteins, which hold unique promise as accessible and reliable biomarkers, offering the potential to improve early diagnosis and patient stratification beyond the capabilities of existing markers [4], [5], [6]. PBC has been increasingly associated with alterations in secretory proteins, which play crucial roles in immune regulation and inflammatory responses. Changes in the expression and secretion of these proteins may contribute to the pathogenesis of PBC and offer new avenues for biomarker discovery and therapeutic targets [7], [8], [9].

Machine learning (ML) approaches offer valuable tools for overcoming challenges inherent in PBC research, such as small sample sizes and the integration of multi-omics data [10]. By applying ML techniques tailored to these constraints, it is possible to uncover complex patterns related to disease progression and treatment response, thereby enhancing biomarker discovery and personalized management strategies [11].

In light of these advancements, there is a pressing need to further elucidate the molecular mechanisms underlying PBC and to validate new biomarkers through large-scale, multi-center studies. Such efforts may not only enhance diagnostic accuracy but also facilitate the identification of novel therapeutic targets, ultimately improving clinical outcomes for patients with PBC [3], 4].

**Figure 1:**
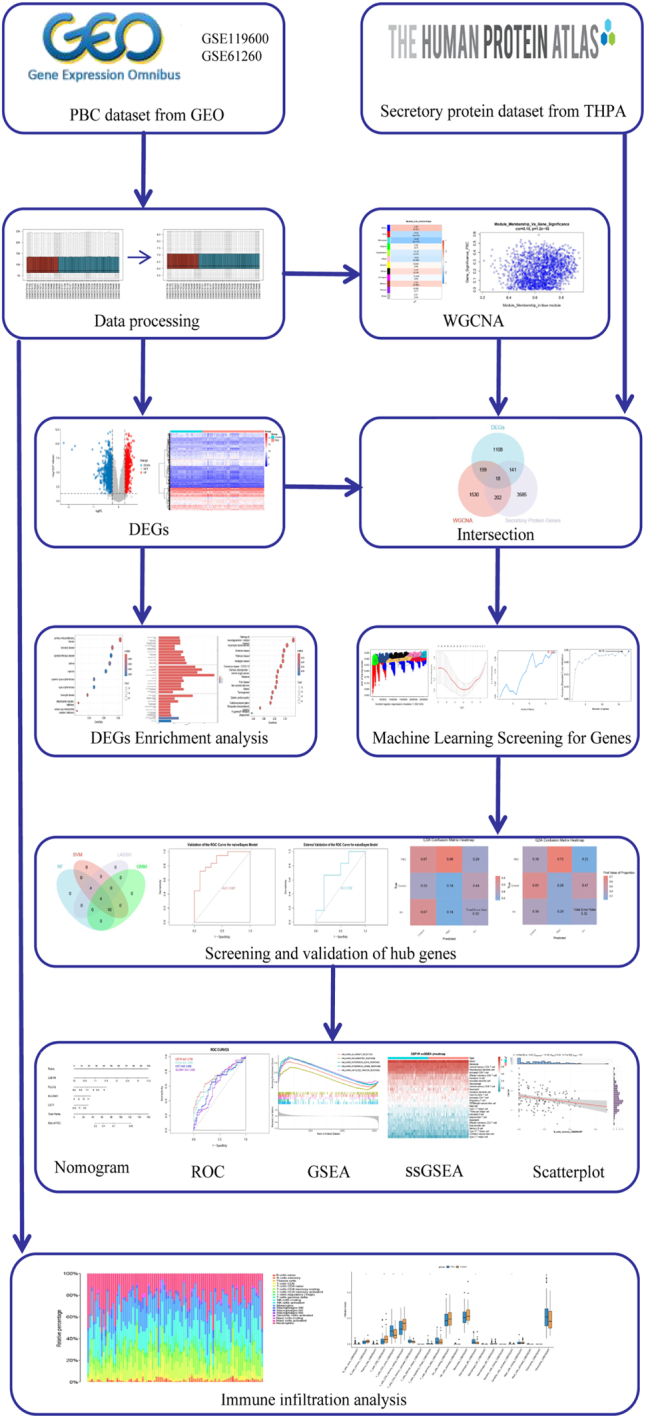
Research flowchart.

Thus, the ongoing exploration of PBC’s pathogenesis and the development of innovative diagnostic and therapeutic strategies are critical. Future research should continue to focus on the integration of omics technologies and advanced computational methods to unravel the complexities of PBC and optimize patient management [4], 11]. The study flow chart is shown in Figure 1.

## 2 Methods and materials

### 2.1 Sources and processing of data

Two PBC-related bulk datasets, GSE119600 and GSE61260, were obtained from the GEO database (https://www.ncbi.nlm.nih.gov/geo/) using the search terms “primary biliary cholangitis” or “primary biliary cirrhosis”. Secreted protein-related gene sets were acquired from the THPA database (https://www.proteinatlas.org/). The datasets included 90 PBC patients and 47 controls from GSE119600, and 11 PBC patients and 38 controls from GSE61260.

### 2.2 Acquisition of differentially expressed genes (DEGs)

GSE119600 was subjected to differential expression analysis using the limma package in R (Ver. 4.3.3), with criteria set to *p* ≤ 0.05 and log2 (fold change) ≥ 0.25, to identify DEGs. The results were visualized using the EnhancedVolcano and heatmap functions.2.3 Differential gene function enrichment analysis.

### 2.3 Differential gene function enrichment analysis

To explore the biological functions and specific mechanisms of PBC-related disease-causing genes, DEGs were subjected to Gene Ontology (GO) function enrichment analysis, Kyoto Encyclopedia of Genes and Genomes (KEGG) function enrichment analysis, and Disease Ontology (DO) disease enrichment analysis, with a threshold of *p* ≤ 0.05 using the clusterProfiler package. The results were presented in bar charts and bubble diagrams.

### 2.4 Weighted gene co-expression network analysis (WGCNA) and hub module gene identification

The association between gene expression in PBC patients and controls was analyzed using weighted co-expression networks. The PBC-control dataset from GSE119600 was used as input data, processed with absolute median deviation treatment, and the co-expression network was constructed using the WGCNA package in R. Sample tree clustering was performed on the input data, and a threshold of 60 was set to exclude outliers. Network topology analysis was conducted to construct a one-step network by setting R^2^ to 0.85 and calculating the soft-thresholding power, followed by the creation of a module cluster graph. The expression patterns of the module genes were assessed in relation to PBC phenotypes using module eigenvalues. Specific modules significantly associated with PBC content were identified through module eigenvector gene analysis. Genes from these modules were selected and intersected with DEGs and secreted protein-related genes to identify candidate genes for PBC diagnosis, with Venn diagrams plotted.

### 2.5 Machine learning

A series of machine learning algorithms were applied to screen hub genes for diagnosing PBC using the GSE119600 dataset. Subsequently, a diagnostic model was constructed and its efficacy validated. A stratified sampling strategy was used to randomly select 80 % of the samples as the training group via the sample function in R. The remaining 20 % formed the validation group, which evaluated the model’s diagnostic effectiveness and checked for overfitting. The GSE61260 dataset was used as an external test set to further assess the model’s performance on independent data. Four machine learning approaches were used for feature selection and model construction. First, LASSO regression (glmnet package) applied L1 regularization with 10-fold cross-validation (family = “binomial”). The optimal *λ* minimizing binomial deviance identified genes with non-zero coefficients. Second, Random Forest-RFE (caret and randomForest) employed leave-group-out cross-validation (10 folds, rfeControl (method = “LGOCV”)) to recursively eliminate features based on Gini importance, optimizing subset size for accuracy. Third, SVM-RFE (msvmRFE) used a linear kernel SVM with 10 elimination iterations (halve.above = 100), selecting the minimal-error feature subset via ten-fold cross-validation. Fourth, Gaussian Mixture Modeling (mclust) evaluated logistic regression AUCs for gene combinations. Clustering of AUC distributions identified the optimal cluster. Model performance was then validated using naive Bayes classifiers (e1071). Receiver Operating Characteristic (ROC) curves were used to quantify diagnostic efficacy across the training, validation, and test sets.

### 2.6 Nomogram

Nomogram models were constructed using the hub genes. Calibration curves were utilized to assess the predictive accuracy of the column-line graph model. The model’s validity for practical clinical applications was evaluated through decision curve analysis (DCA). ROC curves were plotted, and the area under the curve (AUC) was calculated to assess the diagnostic performance of both the candidate genes and the nomogram model.

### 2.7 Immune infiltration analysis

Gene expression data from PBC patients in GSE119600 were extracted, and the CIBERSORT algorithm from the IOBR package in R was used to evaluate the types and numbers of immune cells in PBC. The ggplot2 package was employed to visualize the proportion of immune cells in each sample. The EPIC and quantiseq algorithms were utilized to evaluate and identify hub immune cells in PBC. PBC samples were categorized into high and low expression groups based on whether hub gene expression exceeded the median of all samples. Immune cell heat maps were plotted using the ggboxplot package. The GSVA package conducted a single-sample gene set enrichment analysis (ssGSEA) to compare the infiltration of 28 immune cells in PBC samples. A comparative plot was generated to examine the expression of CSF1R between control and PBC groups. The PBC-control gene expression matrix from the GSE119600 dataset was extracted, and its ROC curve was plotted. GSEA analysis was performed on hub genes, with enrichment plots created. A scatter plot was generated using the ggscatterstats function to illustrate the correlation between immune cell ratios and hub gene expression.

### 2.8 Animal model validation

Twenty mice were randomly divided into two groups, with 10 in the model group and 10 in the control group. The PBC model was established using 2-OA-BSA combined with intraperitoneal injection of PolyI [12]. Following modeling, laboratory animals were euthanized in accordance with ethical and humane guidelines. Euthanasia was conducted using 1 % sodium pentobarbital for intraperitoneal injection, with dosage based on body weight to ensure rapid and painless loss of consciousness. Anesthesia was followed by euthanasia via cardiectomy, ensuring quick cessation of heartbeat and respiration, leading to irreversible loss of brain function. This method minimized unnecessary pain and distress. All personnel involved in euthanasia were professionally trained and qualified. The protocol was reviewed and approved by the Institutional Animal Ethics Committee to ensure ethical and scientific rigor. After euthanasia, a thorough examination was performed by a qualified professional to confirm irreversible death. The study design adhered strictly to the 3R principle, minimizing animal use while maximizing experimental efficiency and quality for scientific integrity and humane practices. The livers from both groups were extracted for HE staining to confirm successful induction of the PBC model. Subsequently, differential genes identified through bioinformatics analysis and machine learning were validated for actual expression using qRT-PCR and Western blot.

Ethical Approval: The research related to animal use has been complied with all the relevant national regulations and institutional policies for the care and use of animals, and has been approved by the Wuhan Hualianke Biotechnology Co., Ltd. and the Animal Ethics Committee (approval number HLK-20230518-002).

## 3 Results

### 3.1 DEGs of PBC

**Figure 2:**
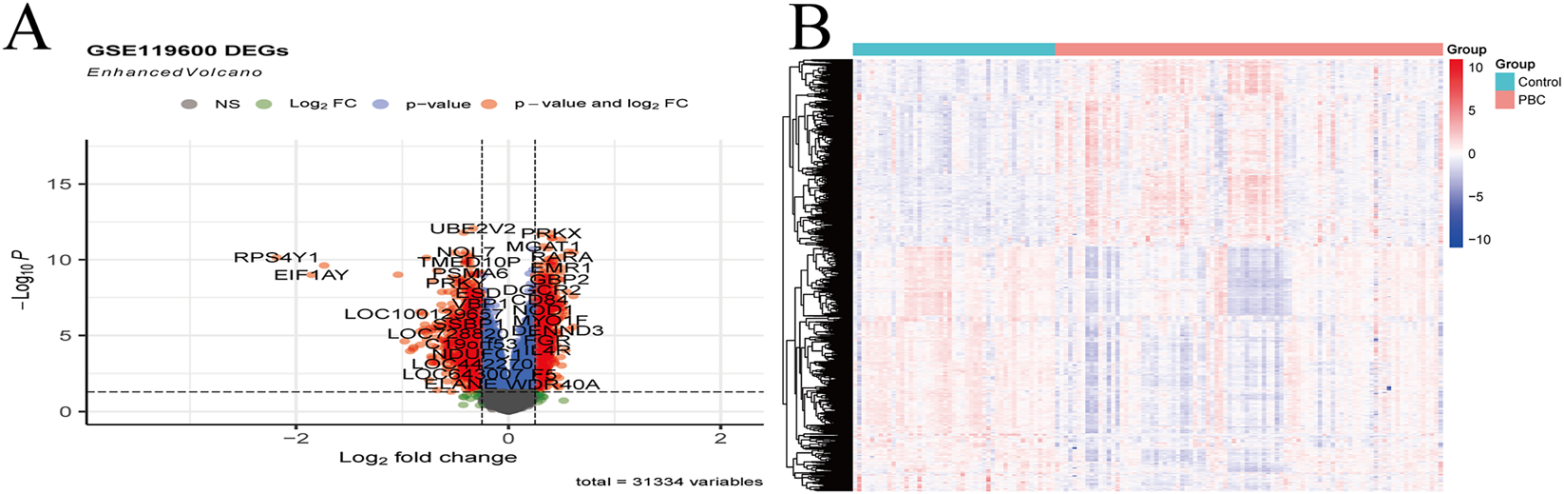
GSE119600 differential gene visualization (A) differential gene volcano map (B) differential gene heat map.

A total of 827 down-regulated genes and 639 up-regulated genes were identified, and their volcano plots were generated (Figure 2A). All DEGs were extracted from the gene expression matrix, categorized into the PBC group and the control group, and visualized in heatmaps (Figure 2B).

### 3.2 Differential enrichment analysis

**Figure 3:**
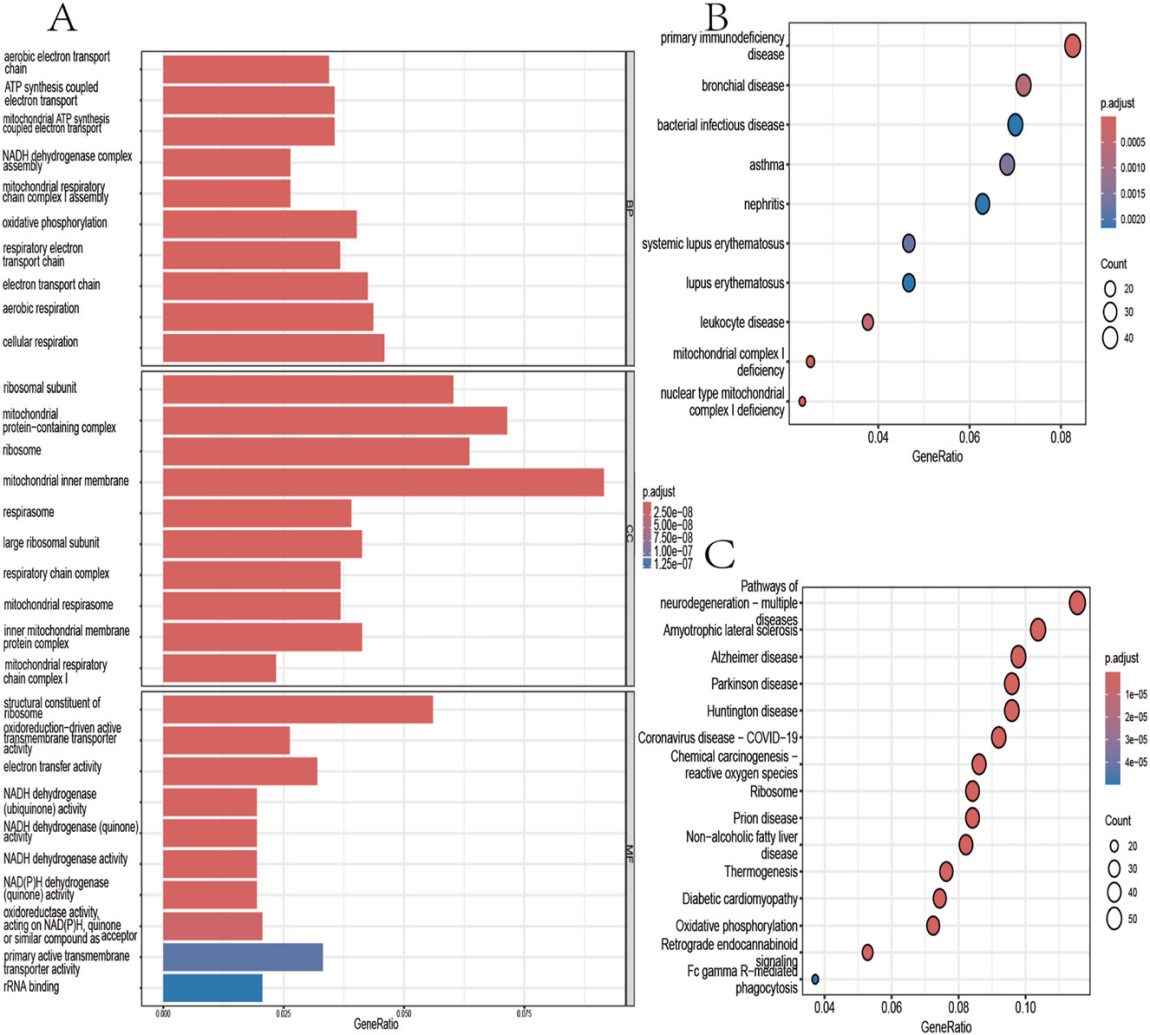
Visualization of differential enrichment analysis (A) GO enrichment analysis (B) DO enrichment analysis (C) KEGG enrichment analysis.

DEGs were enriched in biological processes (BP) such as cellular respiration, aerobic respiration, and the electron transport chain; in cellular components (CC) including the inner mitochondrial membrane, mitochondrial protein complex, and ribosome; and in molecular functions (MF) like ribosome structural composition, primary active transmembrane transporter activity, and electron transfer activity. KEGG analysis indicated that DEGs were significantly enriched in various neurodegenerative diseases, including amyotrophic lateral sclerosis and Alzheimer’s disease. In DO disease analysis, DEGs were significantly enriched in primary immune diseases, bronchial diseases, and bacterial infectious diseases. Visualization results are shown in Figure 3.

### 3.3 Immune infiltration analysis

**Figure 4:**
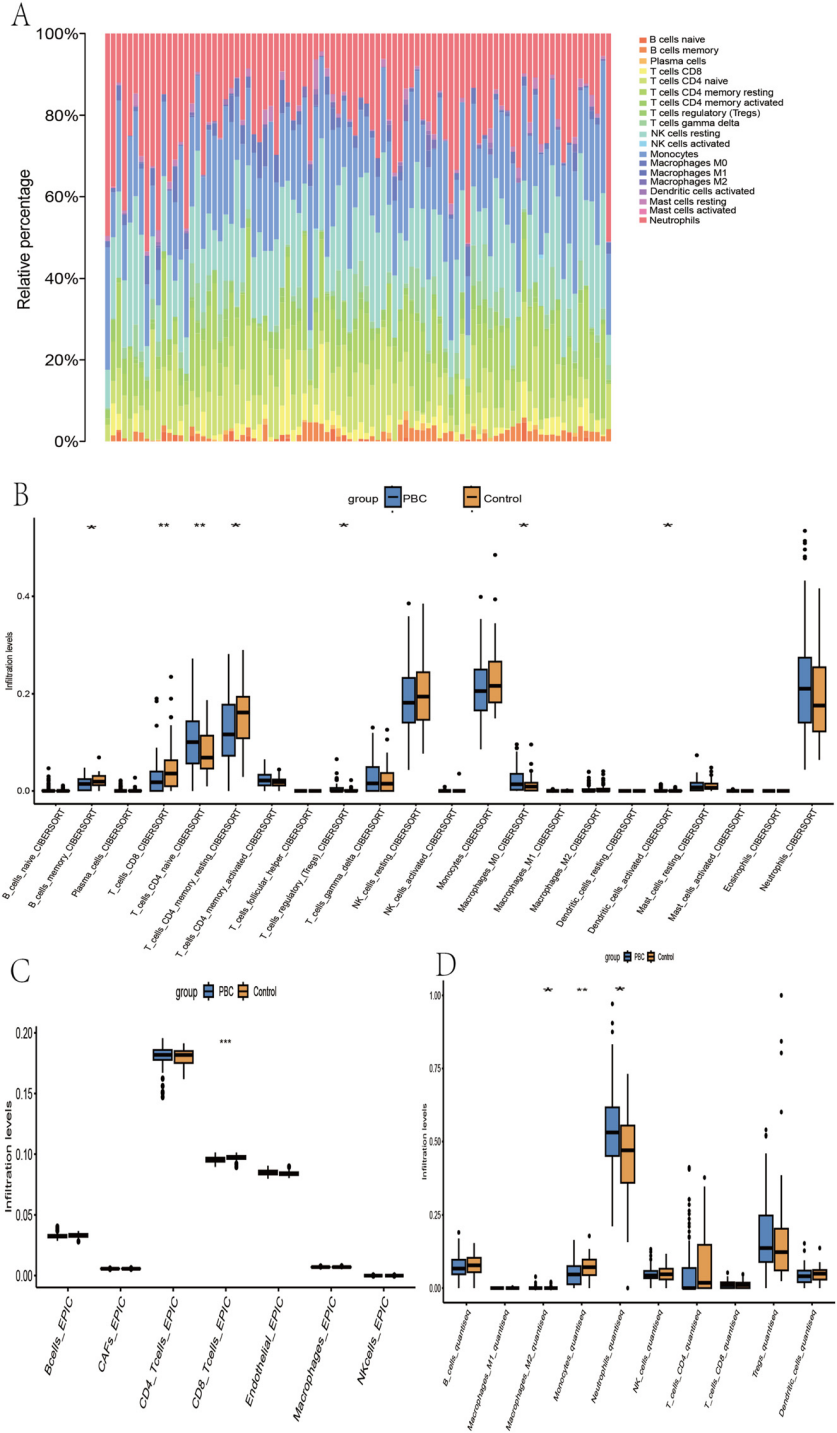
Immuno-infiltration analysis (A) cibersoft PBC scale graph (B) cibersort immuno-infiltration analysis (C) EPIC immuno-infiltration analysis (D) quantiseq immuno-infiltration analysis.

The CIBERSORT algorithm was utilized to evaluate immune cell types in PBC patients and generate a scale diagram (Figure 4A). Immune infiltration analysis was subsequently performed between PBC and control groups using CIBERSORT (Figure 4B), EPIC (Figure 4C), and quantiseq (Figure 4D) algorithms. In the CIBERSORT analysis, memory B cells, CD8+ T cells, resting memory CD4+ T cells, and regulatory T cells were expressed at low levels in PBC patients, whereas immature CD4+ T cells, M0-type macrophages, and activated dendritic cells were highly expressed. The EPIC algorithm also indicated low expression of CD8+ T cells in PBC patients. In the quantiseq algorithm, M2-type macrophages and neutrophils were highly expressed in PBC patients, while monocytes were expressed at low levels.

### 3.4 WGCNA

**Figure 5:**
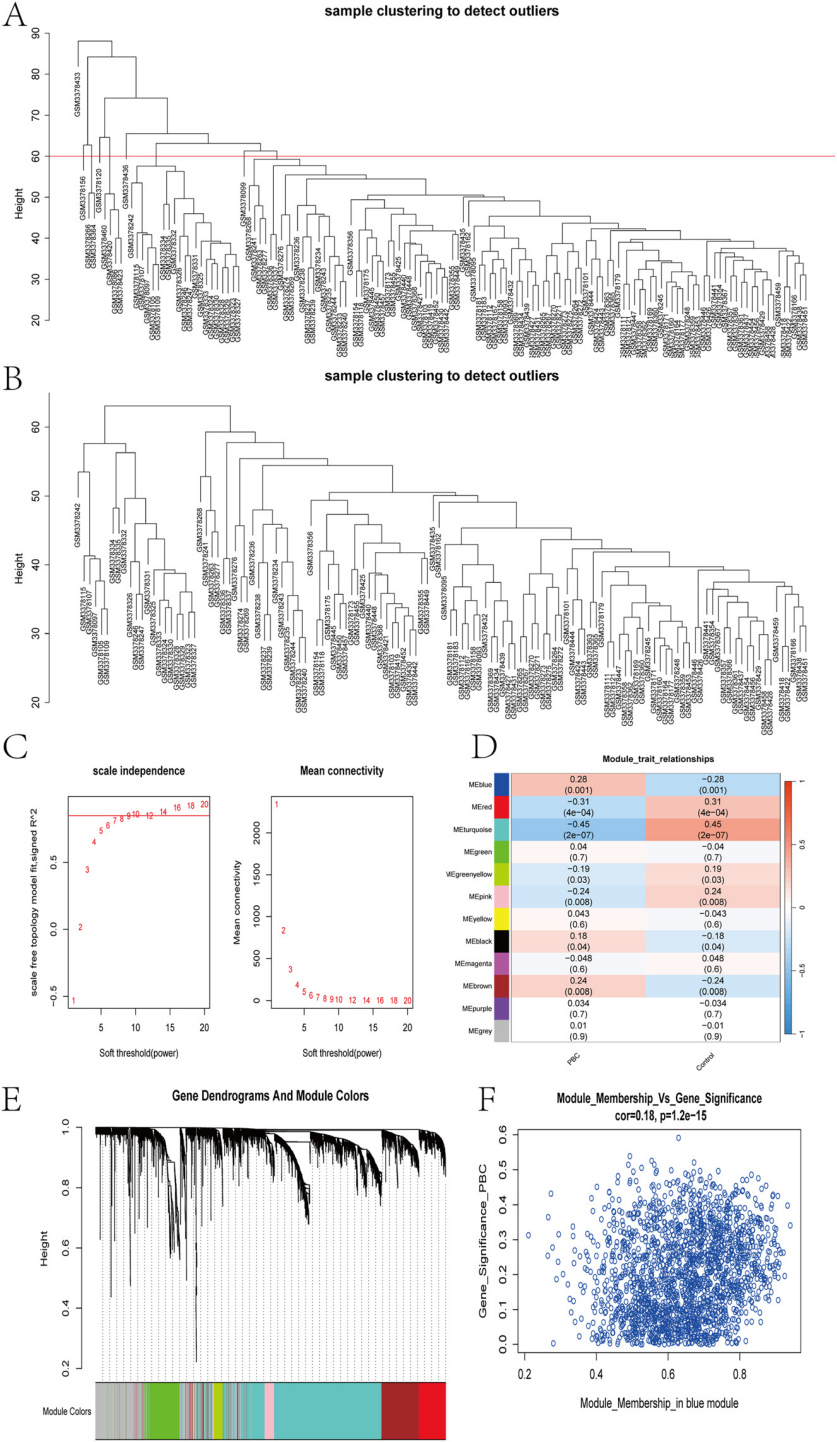
Immuno-infiltration analysis (A) cibersoft PBC scale graph (B) cibersort immuno-infiltration analysis (C) EPIC immuno-infiltration analysis (D) quantiseq immuno-infiltration analysis.

The PBC patients in the GSE119600 dataset were evaluated for scale independence and average connectivity after outlier removal (Figure 5A and B). An optimal power value of 10 was selected based on a correlation coefficient greater than 0.85 (Figure 5C), and the topological overlap matrix (TOM) was constructed with a soft threshold power of 10. A total of 12 gene modules were identified (Figure 5E), and the topological overlap of the gene network is displayed in a heat map (Figure 5D). The correlation value of the blue module with gene significance was 0.18 (*P* < 0.01) (Figure 5F), leading to further studies on genes within the blue module, which contained 1,949 genes.

### 3.5 Result of machine learning

**Figure 6:**
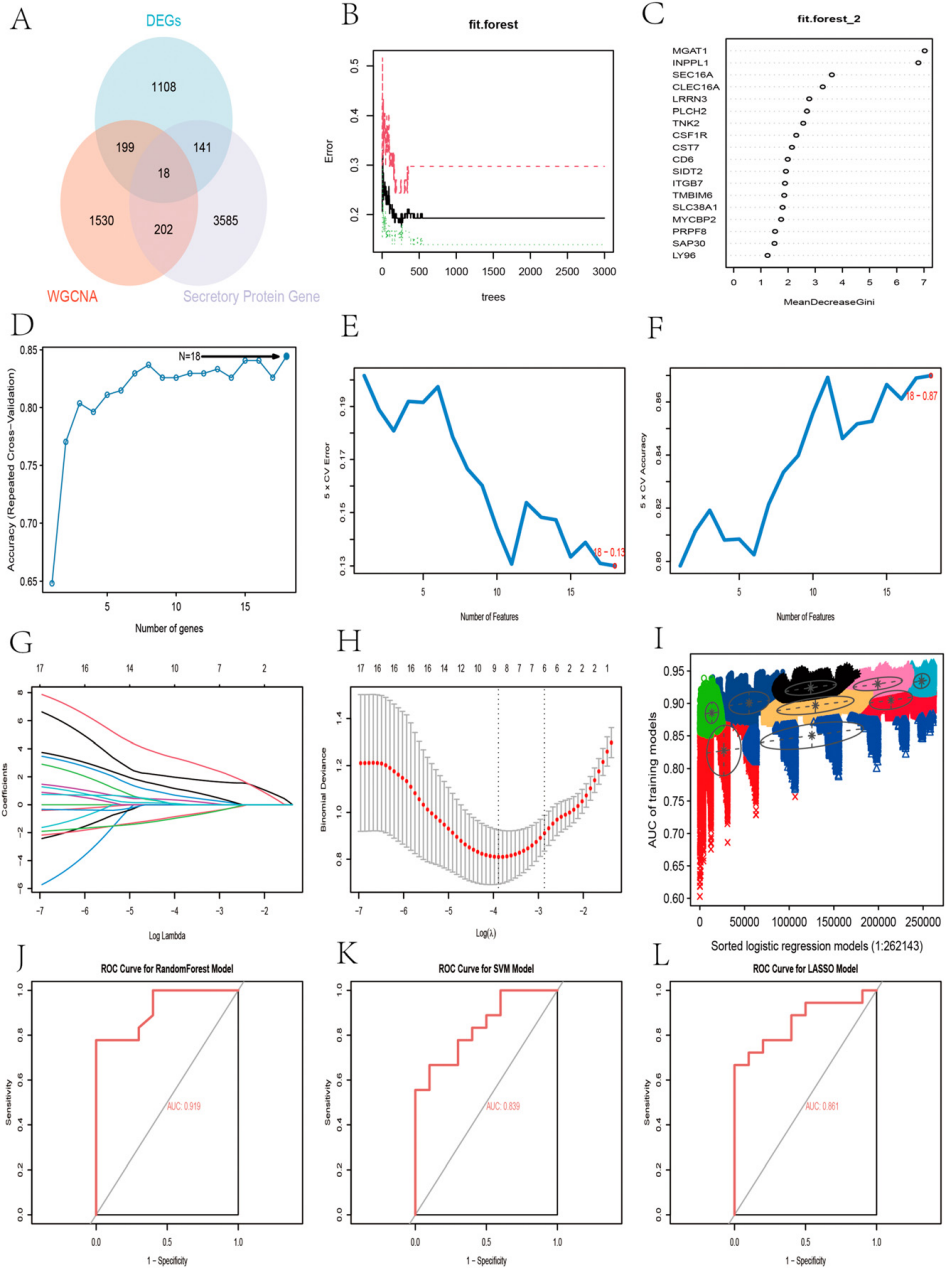
Identification of candidate biomarkers using multiple machine learning algorithms. (A) Venn diagram showing the intersection of DEGs, secreted proteins, and WGCNA genes (B–D) random forest visualization (E–F) SVM-REF visualization (G–H) LASSO visualization (I) GMM visualization (J–L) ROC curves for random forest, SVM-REF, and LASSO.

Candidate genes for diagnosing PBC were identified by intersecting DEGs, secreted protein-related genes, and WGCNA blue module genes, with a Venn diagram plotted (Figure 6A). A series of machine learning analyses were conducted on the expression matrices of the candidate genes to filter out hub genes. First, Random Forest identified 18 candidate genes (Figure 6D), visualizing the prediction accuracy (Figure 6B) and gene importance scores (Figure 6C) of the Random Forest model. Second, SVM-RFE analysis constructed the model, achieving the highest accuracy of 0.87 (Figure 6F) and the lowest error rate of 0.13 with 18 hub genes (Figure 6E). LASSO regression analysis identified 8 hub genes at the lowest lambda value, showing the regression coefficients (Figure 6G) and the results of regression cross-validation (Figure 6H). Finally, GMM analysis identified 14 hub genes at the highest AUC value of the ROC curve (0.96), with individual regression models visualized (Figure 6I). The best models for diagnosing PBC constructed using these machine learning methods were validated with the validation group, with corresponding ROC curves plotted and AUC values calculated (Figure 6J and K, L). The AUC values for Random Forest, SVM-RFE, and LASSO were 0.919, 0.839, and 0.861, respectively.

### 3.6 Expression and ROC curves of hub genes

**Figure 7:**
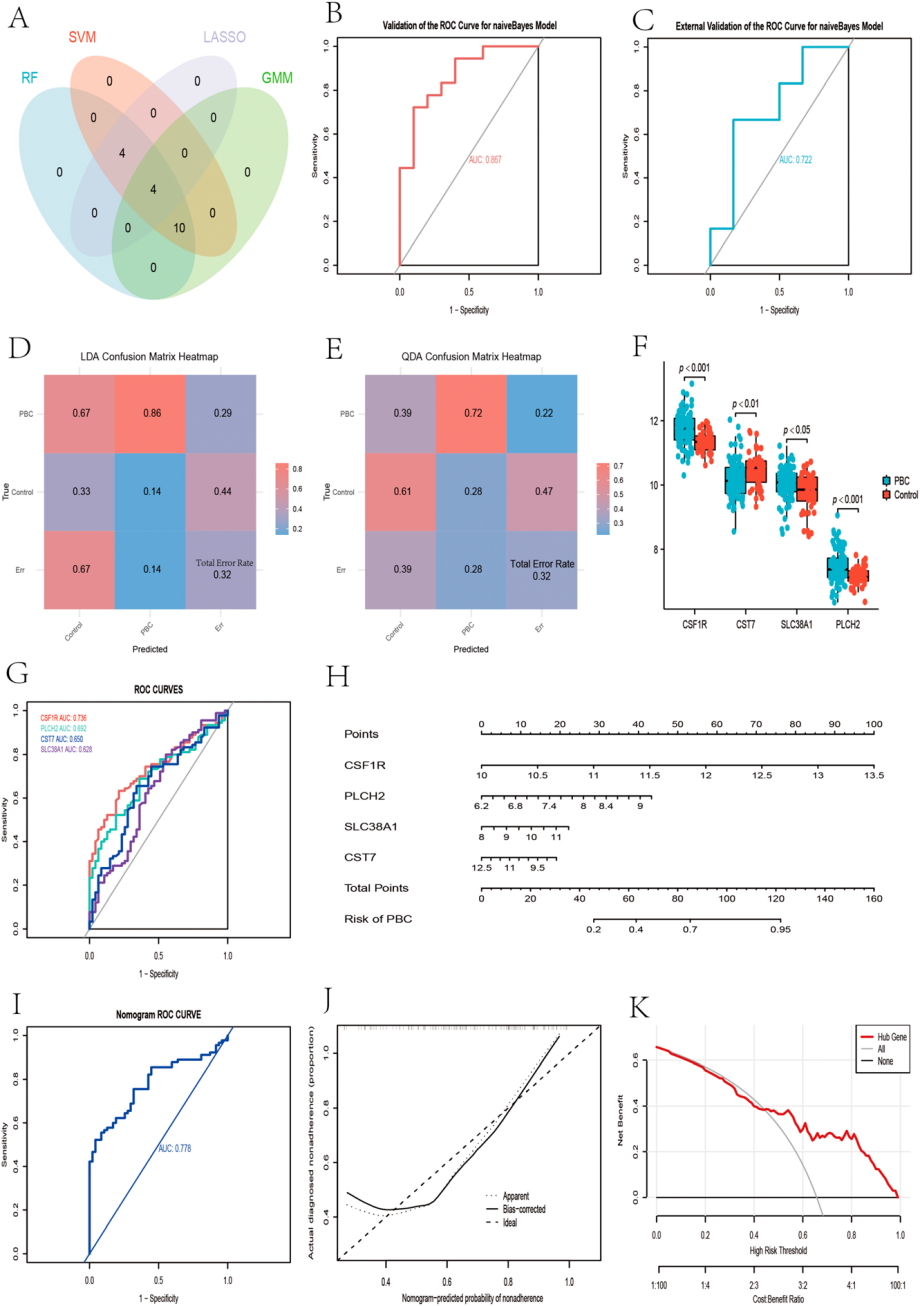
Validation and evaluation of the diagnostic efficacy of hub genes. (A) Venn diagram of machine learning selected genes (B) ROC curve for the validation set of hub genes (C) ROC curve for the test set of hub genes (D–E) heatmaps for LDA and QDA of hub genes (F) comparison plot of hub gene groups (G) individual ROC curves for hub genes (H–K) nomogram visualizations for hub genes.

The hub genes identified by the four algorithms – Random Forest (RF), Support Vector Machine-Recursive Feature Elimination (SVM-RFE), Least Absolute Shrinkage and Selection Operator (LASSO), and Gaussian Mixture Model (GMM) – were intersected to obtain key genes for the diagnosis of primary biliary cholangitis (PBC): CSF1R, PLCH2, SLC38A1, and CST7. The Venn diagrams of these intersections were plotted (Figure 7A). These selected genes were then analyzed using Bayesian inference modeling to evaluate their diagnostic potential. The model’s diagnostic efficacy was assessed using separate validation and test groups. Receiver Operating Characteristic (ROC) curves were plotted, and the area under the curve (AUC) values were calculated for the validation and test groups, yielding 0.867 and 0.722, respectively (Figure 7B and C). Diagnostic error rates were calculated using Linear Discriminant Analysis (LDA) and Quadratic Discriminant Analysis (QDA). Both algorithms showed a total error rate of 0.32. Heat maps were generated to visualize these results (Figure 7D and E).

The expression matrix of hub genes was extracted from the GSE11960 dataset, distinguishing between PBC and control groups. CSF1R, PLCH2, and SLC38A1 were highly expressed in PBC patients, while CST7 was expressed at low levels. Box plots illustrated the differences in hub gene expression (Figure 7F). The diagnostic potency of the respective hub genes for PBC patients in the GSE11960 dataset was calculated, with ROC curves plotted and AUC values calculated (Figure 7G), where CSF1R exhibited the highest AUC at 0.736.

### 3.7 Result of nomogram

A nomogram integrating four hub genes (CSF1R, PLCH2, SLC38A1, and CST7) was constructed to visually translate their expression profiles into individualized PBC risk predictions (Figure 7H). Each gene’s expression level corresponds to a specific point value, and the cumulative score maps to a quantitative probability of PBC. This tool empowers clinicians to estimate disease risk rapidly for individual patients, effectively translating complex genomic data into practical clinical decision-making. To further demonstrate its utility, supplementary analyses were performed: the ROC curve (Figure 7I, AUC = 0.778) quantified the nomogram’s ability to distinguish PBC from controls; the calibration curve (Figure 7J) verified agreement between predicted and actual disease probabilities; and the Decision Curve Analysis (DCA, Figure 7K) demonstrated favorable clinical net benefit across relevant risk thresholds. Together, these results highlight the nomogram’s value as a user-friendly, evidence-supported framework for personalized PBC risk stratification, underscoring its potential to translate genomic insights into clinical practice.

### 3.8 ssGSEA

**Figure 8:**
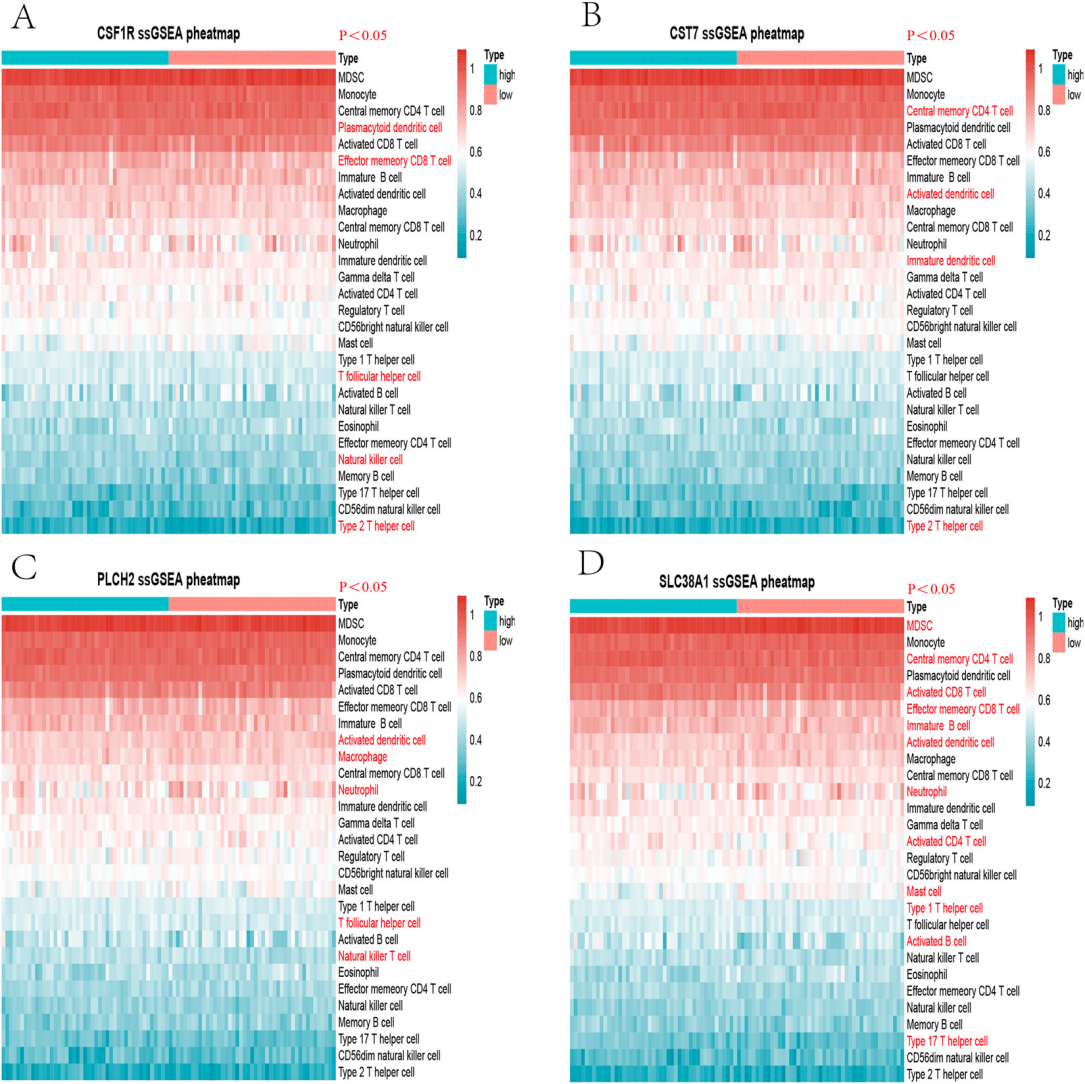
Single-sample gene set enrichment analysis (ssGSEA) of the hub genes. (A) CSF1R ssGSEA heatmap (B) CST7 ssGSEA heatmap (C) PLCH2 ssGSEA heatmap (D) SLC38A1 ssGSEA heatmap.

Immune infiltration analysis using ssGSEA was performed for each hub gene, categorizing PBC patients into high and low expression groups based on whether their expression levels were above the median. The CSF1R high-expression group showed a predominance of effector memory CD8 T cells, natural killer T cells, plasma-like dendritic cells, T follicular helper cells, and type 2 T helper cells compared to the low-expression group. In the CST7 high-expression group, activated dendritic cells were more prevalent than in the low-expression group. Central memory CD4 T cells, immature dendritic cells, and type 2 T helper cells were predominant in the CST7 low-expression group compared to the high-expression group. The PLCH2 high-expression group had more natural killer T cells and T follicular helper cells than the low-expression group, while macrophages, activated dendritic cells, and neutrophils were more prevalent in the PLCH2 low-expression group. The SLC38A1 high-expression group showed more activated B cells, activated CD4 T cells, activated CD8 T cells, central memory CD4 T cells, effector memory CD8 T cells, young B cells, type 1 T helper cells, and type 17 T helper cells than the low-expression group, whereas eosinophils, mast cells, neutrophils, and myeloid-derived suppressor cells were more abundant in the SLC38A1 low-expression group. Results are visualized in heat maps (Figure 8).

### 3.9 CSF1R

**Figure 9:**
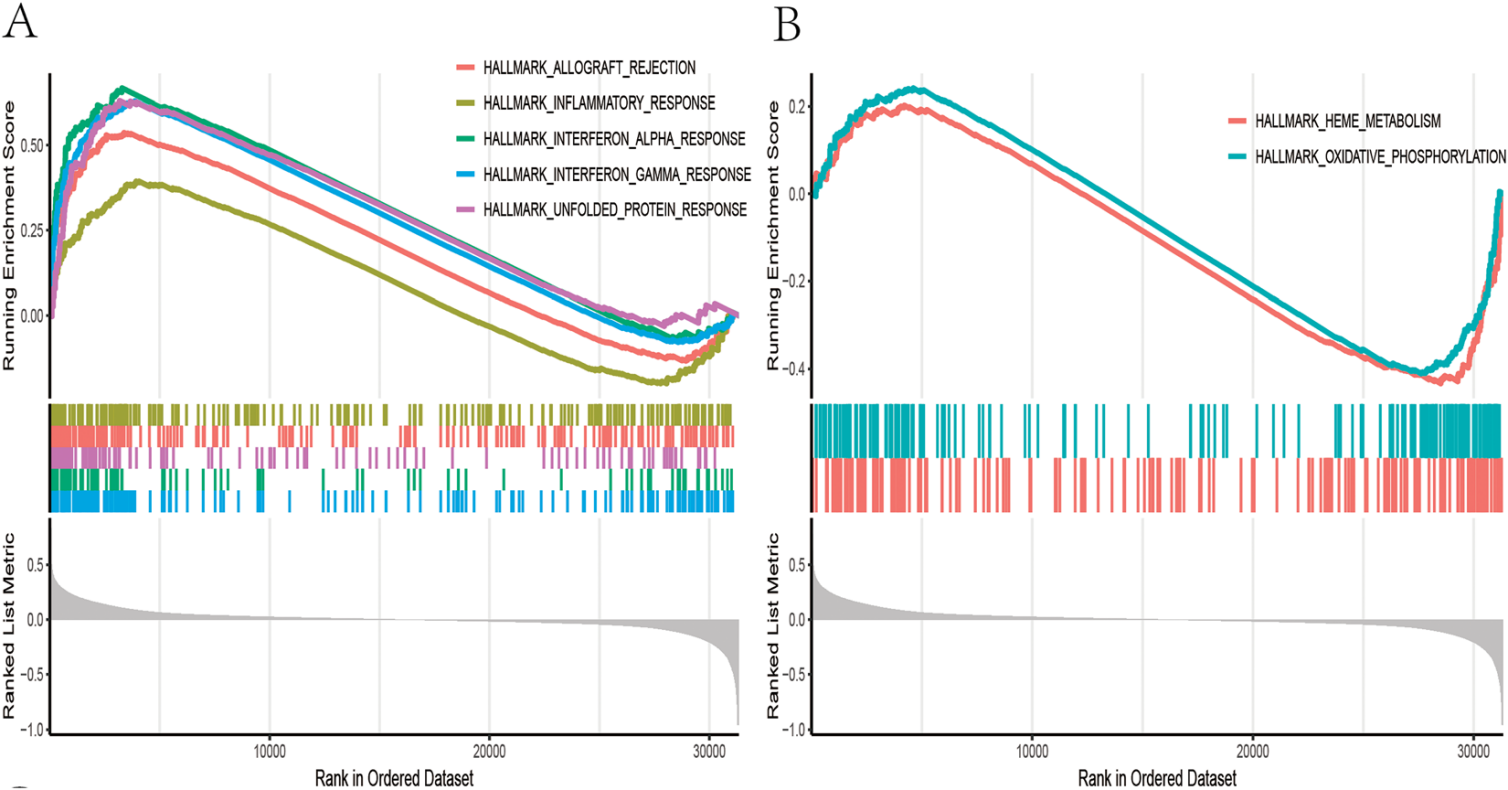
CSF1R GSEA enrichment analysis (A) graph of top five GSEA enrichment analysis shubs for positive NES values (B) graph of GSEA enrichment analysis shubs for negative NES values.

**Figure 10:**
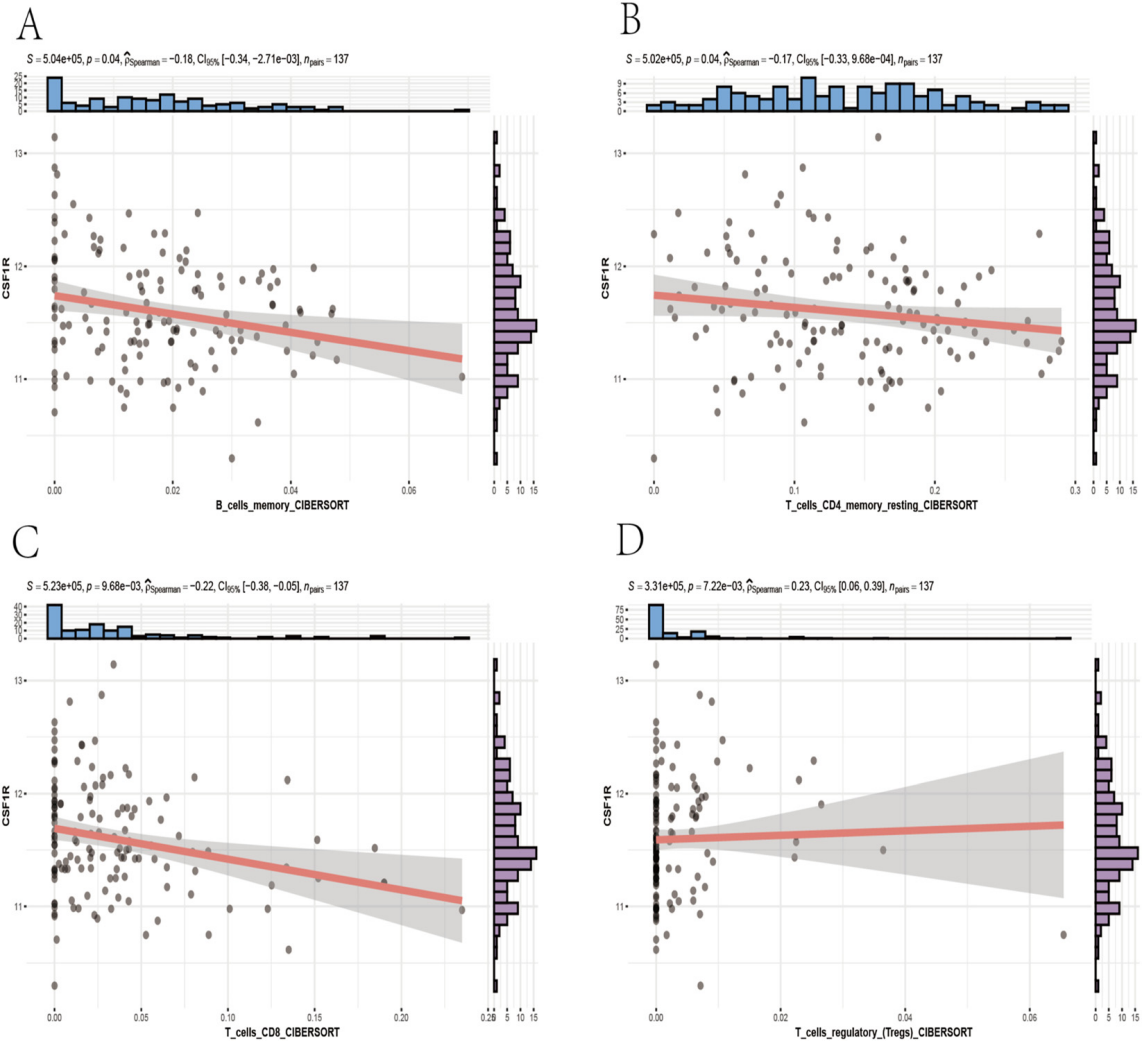
Correlation analysis between CSF1R expression and immune cell infiltration. (A) CSF1R memory B cells immune infiltration scatter plot (B) CSF1R CD4 T cells immune infiltration scatter plot (C) CSF1R CD8 T cells immune infiltration scatter plot (D) CSF1R regulatory (tregs) T cells immune infiltration scatter plot.

The best-expressed CSF1Rs among the hub genes underwent GSEA analysis. PBC patients were divided into CSF1R high and low expression groups based on whether their CSF1R expression exceeded the median, followed by GSEA enrichment analysis using hallmark gene sets. The absolute value of NES was taken as the top five positive values and two negative values for plotting GSEA enrichment analysis plots (Figure 9). The CSF1R high-expression group was mainly enriched in allograft rejection, inflammatory response, interferon *α* response, interferon *γ* response, and unfolded protein response, while the CSF1R low-expression group was primarily enriched in heme metabolism and oxidative phosphorylation. CSF1R gene expression data were extracted, and the results of the CIBERSORT immune infiltration analysis of PBC patients were plotted as immune component correlation scatter plots (Figure 10), indicating significant correlations between CSF1R gene expression and B memory cells, CD4+ memory resting T cells, CD8+ T cells, and regulatory T cell components.

### 3.10 Animal experiment results

**Figure 11:**
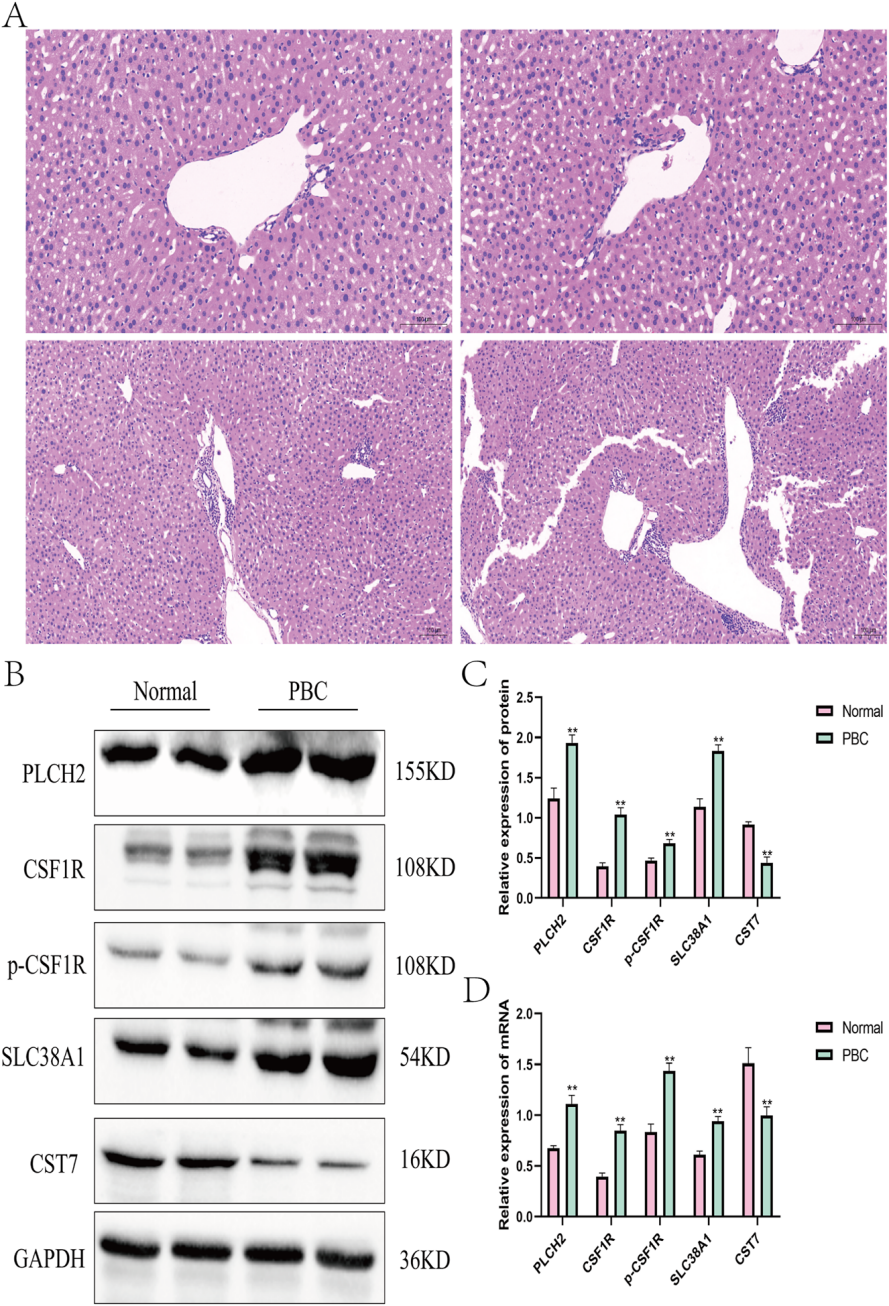
Experimental validation of hub gene expression in vivo. (A) Results of HE staining (B–C) Protein expression levels of PLCH2, CSF1R, p-CSF1R, SLC38A1 and CST7 (D) mRNA expression levels of PLCH2, CSF1R, p-CSF1R, SLC38A1 and CST7 (Compared with the normal group*P < 0.05,**P < 0.01).

HE staining results revealed multiple inflammatory cell aggregates in the hepatic portal vein area of PBC mice. Granulomas were observed within areas of inflammatory cell aggregation, accompanied by extensive lymphocytic infiltration in the biliary epithelium. Western blot and qRT-PCR results demonstrated significantly elevated mRNA and protein levels of PLCH2, CSF1R, *p*-CSF1R, and SLC38A1 compared to the normal group, while CST7 levels were decreased, consistent with previous bioinformatics analysis results (Figure 11).

## 4 Discussion

In this study, a diagnostic model of PBC was developed through the integrated application of bioinformatics analysis and machine learning techniques. Additionally, an in-depth study of its pathogenesis was conducted. The diagnostic model was verified through experiments. CSF1R, SLC38A1, PLCH2, and CST7 together formed the basis of a diagnostic model for PBC, which can be used for the early diagnosis of PBC and facilitate its clinical preventive treatment.

The results of the DO enrichment analysis suggest a potential link between DEGs in PBC and mitochondrial dysfunction. They also indicate associations with autoimmune diseases, inflammatory responses, and abnormalities in immune regulation. Mitochondrial dysfunction may be involved in the pathogenesis of PBC by exacerbating cellular damage and inflammatory responses through mechanisms such as oxidative stress and calcium ion homeostasis imbalance [13], 14]. In addition, the presence of asthma and bronchial diseases further emphasizes the role of chronic inflammation in the pathogenesis of PBC. Inflammatory responses are not only involved in the early course of PBC but may also influence disease progression by promoting the development of hepatic fibrosis and cirrhosis. Inflammatory mediators, such as cytokines and chemokines, play a central role in the pathogenesis of PBC and may be potential targets for future treatment [15], 16].

The results of the GO enrichment analysis showed that PBC is closely related to mitochondrial function. This is especially evident in cellular respiration, the electron transport chain, oxidative phosphorylation, and the assembly and function of the mitochondrial respiratory chain complex I. Impairment of the central role of mitochondria in energy metabolism may lead to decreased energy production, which in turn affects the normal function of liver cells [17], 18].

The results of the KEGG enrichment analyses revealed potential links between PBC and immunomodulation, energy metabolism, inflammatory responses, and neurodegenerative processes. In particular, the enrichment of oxidative phosphorylation pathways highlights the potential role of mitochondrial dysfunction in the energy metabolism associated with PBC. Additionally, pathways involved in diabetic cardiomyopathy and non-alcoholic fatty liver disease may be related to the metabolic disturbances observed in PBC patients [19]. Furthermore, the enrichment of pathways related to reactive oxygen species involved in chemical carcinogenesis may indicate a role for oxidative stress in the pathogenesis of PBC [20], [21], [22]. Although the direct association of pathways in neurodegenerative diseases such as Huntington’s disease, Parkinson’s disease, and Alzheimer’s disease with PBC is not clear, these pathways suggest potential neurological implications of PBC [23], 24].

The results of immune infiltration analysis showed decreased abundance of memory B cells, CD8+ T cells, resting memory CD4+ T cells, and regulatory T cells in patients with PBC, which may indicate suppression of the immune response and imbalance in immune tolerance, potentially promoting the development of autoimmune responses [25], 26]. Meanwhile, the increased abundance of naïve CD4+ T cells, M0-type macrophages, and dendritic cells, as well as an increase in M2-type macrophages and neutrophils, reflects the activation of inflammatory responses and the activated state of immune cells in PBC patients [27], 28]. In addition, the decreased abundance of monocytes may be associated with immunomodulation or immunosuppression in the disease [29].

The CSF1R gene encodes the macrophage colony-stimulating factor 1 receptor (CSF1R), which plays a crucial role in macrophage growth, differentiation, and survival [30]. CSF1R is a transmembrane receptor tyrosine kinase that regulates immune cell function and response by activating downstream signaling pathways, mainly through binding to its ligands CSF1 (M-CSF) or IL-34 [31]. In recent years, numerous studies have shown that CSF1R plays a central role in a variety of immune-related diseases, especially autoimmune and inflammatory diseases [32]. In PBC, CSF1R may significantly impact the onset and progression of the disease by regulating the activities of macrophages and other immune cells [33]. A large number of infiltrating macrophages have been found in the livers of patients with PBC [34]. It remains to be determined whether macrophage activation and survival depend on the CSF1R signaling pathway. Activation of the CSF1R signaling pathway not only promotes macrophage survival and proliferation but also enhances the pro-inflammatory functions of macrophages [35], thereby exacerbating liver inflammation and fibrosis [36].

The PLCH2 (Phospholipase C Eta 2) gene encodes a member of the phospholipase C family, a family of enzymes that plays key roles in intracellular signaling. PLCH2 generates two important second messenger molecules, inositol triphosphate (IP3) and diacylglycerol, by hydrolyzing phosphatidylinositol-4,5-bisphosphate (PIP2), which in turn regulate intracellular calcium ion release and protein kinase C activation, respectively. These processes affect a variety of cellular functions, including cell proliferation, differentiation, and metabolism [37]. Changes in intracellular calcium ion concentration are important regulators of various immune cell functions, including the activation and differentiation of T and B cells [38]. The PLCH2-mediated hydrolysis of PIP2 generates IP3, which induces the release of calcium ions from the endoplasmic reticulum and thereby affects immune cell function [39]. In PBC, aberrant calcium signaling may lead to overactivation of immune cells and excessive production of inflammatory mediators, exacerbating liver injury and fibrosis [40]. PLCH2 influences the pathological process of PBC by regulating the tight junctions and barrier properties of bile duct epithelial cells, thereby affecting their function and barrier integrity [41].

The SLC38A1 (Solute Carrier Family 38 Member 1) gene encodes a sodium-dependent neutral amino acid transporter protein in cells, also known as SNAT1. SLC38A1 is responsible for the transport of a wide range of neutral amino acids, including glutamine, alanine, and leucine, which play important roles in protein synthesis, cell signaling, and energy metabolism [42]. SLC38A1 is expressed in many tissues, particularly in the brain and liver, and its function is critical for the regulation of cellular nutrient uptake and metabolism [43]. By regulating the uptake of key amino acids by immune cells, SLC38A1 may influence their metabolic state and function, which in turn can impact the immune response in PBC [44]. For example, glutamine is an important energy source and precursor for immune cells, and its inadequate supply may impair their activity and function [45]. Additionally, SLC38A1 regulates the uptake of neutral amino acids by hepatocytes and plays a central role in maintaining their metabolic homeostasis and function, thus affecting the progression of PBC [46].

The CST7 gene encodes Cystatin F, also known as Leukocystatin, which is a cysteine protease inhibitor and a member of the Cystatin family of proteins. Cystatin F regulates various physiological processes by inhibiting intra- and extracellular degradation of cysteine proteases, such as cathepsins C and L [47]. These processes include apoptosis, protein degradation, and immune regulation [47]. CST7 is mainly expressed in immune system cells, including natural killer (NK) cells and macrophages, and plays an important role in modulating immune responses and inflammatory processes [48]. Low expression of CST7 may lead to increased cysteine protease activity in the immune system of PBC patients, triggering a series of pathological changes. Elevated cysteine protease activity can cause hyperactivity of immune cells (e.g., NK cells and macrophages) and intensified attacks on bile duct epithelial cells, thereby exacerbating bile duct injury and destruction [49]. Cysteine proteases are crucial in the inflammatory response, and their increased activity may lead to excessive release of pro-inflammatory cytokines and mediators in the liver, further exacerbating inflammation and promoting fibrosis [50].

Recent studies have increasingly highlighted the utility of machine learning in identifying disease-specific biomarkers across autoimmune and hepatic disorders. For instance, Wang et al. employed LASSO and random forest algorithms to identify AKR1B10 as a potential hub gene for PBC [11]. In contrast, our integration of four machine learning approaches, including GMM and SVM-RFE, resulted in a diagnostic model based on CSF1R, PLCH2, SLC38A1, and CST7. Furthermore, CSF1R has been implicated in immune-mediated liver inflammation, particularly in macrophage recruitment and activation, as reported by Wen et al. [34]. Our GSEA results extend this understanding by showing its enrichment in interferon response and unfolded protein response pathways, indicating its potential as not only a diagnostic biomarker but also a therapeutic target in PBC. Notably, CST7 – largely overlooked in hepatic autoimmune diseases – was found to be downregulated in our model, consistent with its role in cysteine protease regulation during chronic inflammation [47], 48]. This novel finding may explain the exacerbated bile duct epithelial injury seen in PBC patients with low CST7 expression. Compared to previous works such as Zhao et al. [26], who focused on general macrophage-driven inflammation, and Pham et al. [10], who applied ML in broader gene set screening, our study narrows down to secretory proteins and establishes their link to immune cell infiltration patterns using multi-method immune deconvolution (CIBERSORT, EPIC, and ssGSEA). This approach offers a more integrated and clinically actionable framework for early diagnosis and immune profiling in PBC. Although our study is focused on secretory protein biomarkers in PBC, similar machine learning methodologies have demonstrated potential in other fields such as neuroinflammation, microbial resistance analysis, and metabolic profiling [51], [52], [53], further reinforcing the cross-domain applicability of computational tools in precision medicine.

## 5 Conclusions

In this study, CSF1R, PLCH2, SLC38A1, and CST7, genes related to secretory proteins, were identified as potential diagnostic markers for PBC using bioinformatics analysis and machine learning techniques. The abnormal expression of these genes may be closely associated with the pathological process of PBC, highlighting their central roles in immune regulation, metabolic homeostasis, and inflammatory response. However, this study has some shortcomings. It includes a relatively small sample size, with external validation relying on the publicly available GSE61260 dataset containing only 11 PBC patients, which limits the statistical power to capture disease heterogeneity and biomarker dynamics. Additionally, validation experiments are insufficient, and further research is needed, including large-scale validation through multicenter collaboration with diverse PBC cohorts covering age, gender, ethnicity, and clinical phenotypes. Such efforts are necessary to translate the bioinformatics-derived results into clinical applications and to clarify the diagnostic efficacy and cut-off values of biomarkers. In the future, in-depth study of the functions and mechanisms of these genes is expected to lead to the development of precise therapeutic strategies for PBC, thereby improving the prognosis and quality of life for patients.
